# The active aging level of the rural older adults with disability in China: a cross-sectional study

**DOI:** 10.3389/fpubh.2023.1219573

**Published:** 2023-08-01

**Authors:** Yutong Tian, Yan Zhang, Yuwen Yan, Huizhong Zhang, Xizheng Li

**Affiliations:** School of Nursing and Health, Zhengzhou University, Zhengzhou, Henan, China

**Keywords:** aging, older adults, disability, active aging, health aging, quality of life

## Abstract

**Background:**

Active aging has been listed as an important indicator to measure the quality of life of the older adults and the construction of the senior care system. There is an imbalance between the supply and demand of senior care services for the disabled older adults in rural areas, and the quality of life needs to be improved.

**Objectives:**

We aimed to analyze the current situation of active aging and the influencing factors of the rural disabled older adults, in order to provide a reference basis for improving the quality of life of the rural disabled older adults.

**Methods:**

We conducted a multicenter and cross-sectional study, using the Barthel Index Scale and Chinese version of the Active Aging Scale, to facilitate the selection of 304 rural older adults with disability in 26 villages under Henan Province for a questionnaire survey.

**Results:**

The mean score for the level of active aging of rural older adults with disability was 1.87 (SD 0.36), with the highest score for the dimension of being self-reliant (Mean2.29, SD 0.61) and lower scores for the dimension of active contribution to society (Mean 1.37, SD 0.55) and building up financial security (Mean 1.37, SD 0.57). The results of the multiple regression analysis showed higher levels of active aging among the disabled older adults with retirement pay, mild disability, and longer time per activity/rehabilitation exercise (*p* < 0.05).

**Conclusion:**

Active aging of the rural disabled older adults is at a low level, with insufficient economic security and social participation. The national government should help improve the quality of primary health care in rural areas, build a friendly environment for senior communities, and improve policies to protect the welfare of the older adults, so as to collaboratively empower the disabled older adults in rural areas at three levels: health, participation, and protection.

## 1. Introduction

The number and proportion of older people in countries around the world is on the rise, with the number of people over 60 increasing from 1 billion to 1.4 billion between 2020 and 2030, and the number of people over 60 expected to increase to 2.1 billion in 2050, and the number of people over 80 tripling to 426 million, with the greatest trend of change in low-and middle-income countries (1). China’s seventh census data show that by 2020 China’s older adults aged 60 and above has reached 264 million (2), the scale of disabled older adults has reached 6.18 million, the proportion of disabled older adults in rural areas reached 50.16%, the disablement rate of rural older adults reached 2.86%, which is 0.58% higher than that in urban areas (3), the phenomenon of aging and disablement in rural areas is serious and has gradually become the strategic focus of the country to actively deal with the problem of aging population.

In 2002, the World Health Organization officially introduced the concept of active aging and defined active aging in its Global Report on Aging and Health as “the process of optimizing the health, social participation and security opportunities of older adults in order to improve their quality of life (4). Active aging has been recognized by the international community as a fundamental initiative to address population aging (5). The European Union, as the regional international organization that has used and studied the concept of active aging the most and the earliest, has further validated the feasibility and socio-economic benefits of active aging strategies by transforming policy advocacy into concrete policy actions based on coordination among member states (6). In addition, establishing the perception of active aging has been proven to improve physical function, cognitive function, mental health, social health and sleep status of older adults (7).

In 2017, China issued the “13th Five-Year Plan for the Development of the National Aging Career and the Construction of the Pension System,” which listed active aging as an important indicator for measuring the quality of life of the older adults and the construction of the pension system (8). In 2021, the “Outline of the 14th Five-Year Plan for National Economic and Social Development and Vision 2035” established the national strategic position of actively coping with population aging (9). In order to implement this national strategy, the “Opinions on Strengthening the Work on Aging in the New Era” issued by the Central Committee of the Communist Party of China and the State Council put forward a number of macroscopic guidelines on the senior care service system, health support system, social participation of the older adults and silver hair economy, and emphasized the need to integrate the concept of active aging and healthy aging into the whole process of economic and social development, advocate the implementation of mutual aid and medical and nursing care models, and strengthen long-term care services and protection for the disabled older adults (10). However, there are still many dilemmas in the actual operation of the combined medical and nursing care and mutual care models in rural China, such as incomplete integration of resources, lack of nursing professionals, and difficulties in building organizational support (11, 12). Additionally, multiple factors such as lack of medical care, financial difficulties, age discrimination and social exclusion also seriously hinder the development of active aging in rural areas (13).

Health, participation, and security are the three pillars of active aging. Rural older adults with disability have declining physical functions, lack of health knowledge, cognitive bias, weak awareness, limited social and productive participation (14), lack of adequate protection of their rights and interests in material economy, medical assistance, spiritual and cultural life (15), and low level of well-being and self-assessed health (16). However, the rural older adults with disability have strong needs in terms of health knowledge, social participation, and social security, and a comprehensive assessment is urgently needed to improve their active aging (17). Currently, there is an increasing number of studies on active aging, which mainly focus on policy exploration, pathways and multidimensional factor analysis at the theoretical level (18, 19), and related intervention studies are mostly focused on older adults in urban communities, but less benefit older adults in rural and remote areas, and only the research team has conducted a qualitative study focusing on the level of active aging of older adults with disability in rural areas (17). Therefore, this study combines the qualitative research results of the previous research group and adopts quantitative thinking to investigate the current status of active aging of rural disabled older adults on a large scale, in order to provide a reference basis for improving the rural senior care service system.

## 2. Materials and methods

### 2.1. Study design and participants

This study used a multicenter and cross-sectional survey research design. From July to December 2022, a random number table method was used to select three prefectures from 17 prefectures in Henan Province, China, as the study site, and subsequently to facilitate the selection of questionnaires from 26 administrative villages under them for the older adults with disability. Inclusion criteria: ① ≧60 years old; ② Have rural household registration in China and have lived in the local area for at least 1 year; ③ Barthel index (BI) assessment <100 points, those with partial or total loss of self-care ability; ④ No serious visual–auditory or communication dysfunction; ⑤ Informed consent and voluntary participation in this study. Disabled rural older adults with severe mental illness or cognitive dysfunction who were unable to cooperate with the survey were excluded. We used the empirical method to calculate the sample size, which involved a total of 19 demographics variables and 7 active aging variables in this study, taking 5–10 times the number of variables, and then considering a 10–20% missed visit rate, the calculated sample size range was 144–325.

### 2.2. Measures

#### 2.2.1. Demographics

Participants reported their gender, age, ethnicity, religion, education level, marital status, number of children, residence status, economic source, monthly income (RMB), medical expenses/month, form of medical insurance, level of disability, length of disability (years), and reason for disability. The researchers also collected the status of rural health activity facilities, the number and length of activity/rehabilitation exercises for the disabled older adults, and the degree of family support for the disabled older adults to participate in social activities.

#### 2.2.2. Barthel Index Scale

The Barthel index (BI) scale consists of 10 items: eating, bathing, grooming, dressing, bowel control, urinary control, toileting, bed and wheelchair transfer, level walking, and stair climbing, and is scored out of 100 according to the degree of assistance the patient needs to complete each item (independent, partially assisted, extremely assisted, and totally dependent). Rural older adults with disability are classified into three levels based on BI scores: 61–99 mild disability, 41–60 moderate disability, and 0–40 severe disability. The BI scale is simple to use and has high sensitivity and reliability, with a retest reliability of 0.89 and inter-rater reliability greater than 0.95, and has been widely used to assess patients’ ability to perform activities of daily living (20).

#### 2.2.3. Active Aging Scale

The Active Aging Scale (AAS) was developed by Thai scholar Thanakwang et al. (21) based on the theory of active aging, and research team member Jiange et al. (22) used the Brislin translation model (23) to translate, back-translate, and culturally adapt the scale based on the authorization of the original authors, and revised the item presentation by combining the results of cognitive interviews with rural older adults to form the Chinese version of the Active Aging Scale, which is used to measure the level of active aging of rural older adults with disability. AAS contains 7 dimensions: being self-reliant (7 items), active learning and social integration (8 items), growing spiritual wisdom (5 items), building up financial security (4 items), maintaining healthy lifestyle (5 items), active contribution to society (4 items), and passing on filial piety by example (3 items). 36 items were evaluated on a 4-point Likert scale with a total score range of 36–144, with higher scores indicating higher levels of active aging. The Chinese version of the AAS has good reliability and validity, the scale content validity index (S-CVI/Ave) is 0.981, the content validity index of each item (I-CVI) is 0.83 ~ 1.00, the Cronbach’s alpha coefficient is 0.932, the Cronbach’s alpha coefficient of each dimension is in the range of 0.777–0.913, and the retest reliability is 0.725.

### 2.3. Data collection

Data collection was conducted by three graduate students (YTT, YWY, HZZ) trained in uniform questionnaire distribution, using on-site distribution of paper versions of the questionnaires. First, the research team mentor (YZ) contacted the township health centers under the three local municipalities to obtain support and informed consent, and then entered each rural area under the leadership of a member of the township health center, accompanied by a village doctor to conduct a questionnaire survey in the households. Then we introduced the purpose and method of the study to the rural older adults with disability and distributed the questionnaires on site. The graduate students uniformly read the questionnaire entries to the older adults with disabilities in a neutral manner and assisted them in checking the boxes, which were collected immediately after completion, and all questionnaires were surveyed anonymously. After 5 months of survey, a total of 308 questionnaires were returned, of which 304 were valid, with a valid questionnaire return rate of 98.7%.

### 2.4. Statistical analysis

We conducted all data entry and statistical analysis using SPSS 25.0 (SPSS Inc., Chicago, IL, USA), with categorical data described by frequency and percentage and continuous data described by mean and standard deviation (SD). We also reported the minimum, maximum, and 95% confidence intervals for the mean of each dimension score. Additionally, we applied Q-Q plots to test for normality and the results showed that the outcome variable data points were normally distributed around the diagonal. Two independent samples t-test was used to compare the means of two groups, and one-way ANOVA was used to compare the means of three or more independent samples, and Bonferroni method was chosen for chi-square and Tamheni method and reference Welch test correction results were chosen for chi-square. Furthermore, we used multiple linear regression for impact factor analysis with a significance level of 0.05.

## 3. Results

### 3.1. Participants’ characteristics

The 304 rural older adults with disabilities ranged in age from 60 to 92 (Mean 71.84, SD 8.23) years, BI score from 10 to 95 (Mean 69.72, SD 19.02) and length of disability from 1 to 40 (Mean 6.17, SD 5.40) years, and all of the disabled older adults suffered from different conditions of chronic diseases, in order of prevalence: hypertension (203/66.8%), neurological diseases (181/59.5%), joint pain (125/41.1%), diabetes mellitus (57/18.8%), coronary heart disease (41/13.5%), cataract (23/7.6%), gastritis (20/6.6%), respiratory diseases (12/3.9%), hyperlipidemia (9/3.0%), etc., with specific demographic information shown in Table 1.

**Table 1:** Intergroup comparison of active aging level of rural disabled older adults.

Characteristics	*n*(%)	Mean	SD	*F*/*Z*	*P*
Gender				−0.703^*^	0.482
Male	156 (51.3%)	67.69	13.80			
Female	148 (48.7%)	67.05	12.39				
Age				1.771^**^	0.156
60~	66 (21.7%)	68.44	9.77			
65~	132 (43.4%)	65.67	11.89				
75~	49 (16.1%)	67.47	17.61				
80~	57 (18.8%)	70.00	14.42				
Ethnicity				−0.418^*^	0.616
Han ethnic group	298 (98.0%)	67.42	13.23			
Ethnic minorities	6 (2.0%)	65.00	3.10				
Religion				0.234^*^	0.815
None	224 (73.7%)	67.29	13.73			
Yes	80 (26.3%)	67.61	11.29				
Education level				5.145^**^	0.006
Primary school and below	257 (84.5%)	66.36	13.20			
Junior high school	44 (14.5%)	73.00	11.27				
High school and above	3 (1.0%)	72.00	12.12				
Marital status				0.430^**^	0.731
Unmarried	4 (1.3%)	68.75	7.09			
Married	220 (72.4%)	67.57	12.84				
Divorced	5 (1.6%)	61.00	8.22				
Widowed	75 (24.7%)	67.15	14.39				
Number of children				0.381^**^	0.767
0	7 (2.3%)	64.57	7.23			
1	14 (4.6%)	66.57	15.01				
2	78 (25.7%)	66.38	11.86				
3~	205 (67.4%)	67.90	13.62				
Residence status				3.302^**^	0.011
Living with children	57 (18.8%)	63.67	14.71			
Living with spouse	109 (35.9%)	66.19	12.06				
Living with children and spouse	209 (35.9%)	69.51	13.21				
Living alone	7 (2.3%)	77.43	6.27				
Nursing home	22 (7.2%)	69.05	11.90				
Economic sources				23.882^**^	<0.001
Retirement pay	23 (7.6%)	86.30	10.10			
Child support	88 (28.9%)	64.58	13.95				
Government relief	48 (15.8%)	63.90	11.16				
Pension	113 (37.2%)	65.61	9.39				
Other (part-time work, farming, etc.)	32 (10.5%)	72.91	14.32				
Monthly income (RMB)				22.899^**^	<0.001
<500	193 (63.5%)	65.56	11.30			
500~	94 (30.9%)	67.64	13.91				
1000~	17 (5.6%)	86.53	12.94				
Medical expenses/month				4.570^**^	0.012
<100	69 (22.7%)	66.41	12.42			
100~	160 (52.6%)	69.36	13.30				
500~	75 (24.7%)	64.03	12.71				
Medical insurance form				−2.301^*^	0.021
Urban employee medical insurance	7 (2.3%)	78.00	10.94			
Urban and rural residents’ medical insurance	297 (97.7%)	67.12	13.07				
Disability level				107.139^**^	<0.001
Mild disability	219 (72.0%)	71.06	11.74			
Moderate Disability	46 (15.1%)	63.15	12.40				
Severe Disability	39 (12.8%)	51.64	6.61				
Length of disability (years)				11.059^**^	<0.001
1~	150 (49.3%)	70.36	13.20			
5~	61 (20.1%)	63.75	14.24				
10~	51 (16.8%)	60.33	10.81				
15~	27 (8.9%)	68.41	6.36		
Reason for disability				0.524^**^	0.596
Disease	247 (81.3%)	66.99	12.15			
Accident	24 (7.9%)	68.96	21.84				
Aging	33 (10.9%)	69.12	11.97				
Number of activities/rehabilitation exercises				33.711^**^	<0.001
Never	113 (37.2%)	58.92	11.42			
Once a month	36 (11.8%)	65.44	7.27				
2 ~ 4 times a month	28 (9.2%)	68.79	8.04				
2 ~ 3 times a week	67 (22.0%)	76.54	10.05				
More than 4 times a week	60 (19.7%)	73.57	13.67				
Activity/rehabilitation exercise hours/time				32.814^**^	<0.001
Never	113 (37.2%)	58.92	11.42			
Under 30 min	123 (40.5%)	68.98	9.37				
30 min~	39 (12.8%)	74.10	9.44				
1 h~	17 (5.6%)	84.12	11.40				
2 h~	12 (3.9%)	84.92	15.64				
Degree of family support for the disabled older adults to participate in social activities				25.837^**^	<0.001
Highly unsupportive	14 (4.6%)	49.00	9.55			
Unsupportive	33 (10.9%)	63.67	10.54				
Normal	98 (32.2%)	61.92	9.24				
Supportive	116 (38.2%)	72.89	12.40				
Highly supportive	43 (14.1%)	73.77	13.82				

^*^Z-value; ^**^F-value.

### 3.2. The scores of active aging level of rural disabled older adults

The mean score of the active aging level of the rural disabled older adults was 67.38 (SD 13.11) and the mean score of the items was 1.87 (SD 0.36), which was at a low level, and the specific scores of each dimension are shown in Table 2.

**Table 2:** Total and average scores of active aging level of rural disabled older adults.

Active aging level	Maximum points	Minimum points	Total scores		Average scores		95% CI	
			Mean	SD	Mean	SD	Lower border	Upper border
Being self-reliant	26	7	16.04	4.24	2.29	0.61	15.56	16.52
Active learning and social integration	23	8	12.92	3.64	1.61	0.45	12.51	13.33
Active contribution to society	15	4	5.49	2.19	1.37	0.55	5.24	5.74
Growing spiritual wisdom	16	5	10.02	2.83	2.00	0.57	9.70	10.34
Building up financial security	16	4	5.50	2.27	1.37	0.57	5.24	5.75
Maintaining healthy lifestyle	20	5	10.81	3.02	2.16	0.60	10.46	11.15
Passing on filial piety by example	11	3	6.60	1.43	2.20	0.48	6.44	6.76

### 3.3. Intergroup comparison of active aging level of rural disabled older adults

There was a statistical difference in the scores of active aging level of rural older adults with disabilities by “education level, residence status, economic source, monthly income (RMB), medical expenses/month, medical insurance form, disability level, length of disability (years), number of activities/rehabilitation exercises, length of activities/rehabilitation exercises/time, and degree of family support for the disabled older adults to participate in social activities”(*p* < 0.05), as shown in Table 1. The results of the two-by-two comparison between groups showed that the active aging level of the disabled older adults in primary school and below was lower than that of those in junior high school, those living with children were lower than those living with children and spouses and those living alone, those living with spouses were lower than those living alone, and those on government assistance were lower than those on other (working, farming, etc.) economic incomes (*p* < 0.05). The higher the monthly income and the lower the degree of disability, the better the active aging level of the older adults, those with retired salary were higher than those with other economic sources, those with monthly medical expenses of 100 or more were significantly higher than those with 500 or more, those with length of disability of 1 year or more were significantly higher than those with 5 and 10 years or more, and those with 15 years or more were significantly higher than those with 10 years or more (*p* < 0.05). The level of active aging was significantly higher for the disabled older adults with activity/rehabilitation exercise 2–3 times per week than for those who never, 1 time per month, and 2–4 times per month, and for those with activity/rehabilitation exercise 1 h or more than for those who never, less than 30 min, and 30 min or more (*p* < 0.05).

### 3.4. Influencing factor analysis of active aging level of rural disabled older adults

Taking the score of active aging level of rural disabled older adults as the dependent variable, and taking the statistically significant variables in the comparison between groups as the independent variables, multiple linear regression analysis was performed after assigning values to the independent variables, as shown in Table 3. The results showed that the four independent variables “economic source, disability level, activity/rehabilitation exercise hours/time” entered the regression equation (*F* = 23.596, *R* = 0.773, *R*^2^ = 0.597, DW = 1.815, VIF < 10), see Table 4 for details.

**Table 3:** Independent variable assignment.

Independent variable	Assignment method
Education level	1 = Primary school and below, 2 = Junior high school, 3 = Junior high school
Residence status	Dummy variables were set using “living with children” as the control group: Living with children (*Z*_1_ = 0, *Z*_2_ = 0, *Z*_3_ = 0, *Z*_4_ = 0), Living with spouse (*Z*_1_ = 1, *Z*_2_ = 0, *Z*_3_ = 0, *Z*_4_ = 0), Living with children and spouse (*Z*_1_ = 0, *Z*_2_ = 1, *Z*_3_ = 0, *Z*_4_ = 0), Living alone (*Z*_1_ = 0, *Z*_2_ = 0, *Z*_3_ = 1, *Z*_4_ = 0), Nursing home (*Z*_1_ = 0, *Z*_2_ = 0, *Z*_3_ = 0, *Z*_4_ = 1)
Economic sources	Dummy variables were set using “retirement pay” as the control group: Retirement pay (*Z*_1_ = 0, *Z*_2_ = 0, *Z*_3_ = 0, *Z*_4_ = 0), Child support (*Z*_1_ = 1, *Z*_2_ = 0, *Z*_3_ = 0, *Z*_4_ = 0), Government relief (*Z*_1_ = 0, *Z*_2_ = 1, *Z*_3_ = 0, *Z*_4_ = 0), Pension (*Z*_1_ = 0, *Z*_2_ = 0, *Z*_3_ = 1, *Z*_4_ = 0), Other (part-time work, farming, etc.) (*Z*_1_ = 0, *Z*_2_ = 0, *Z*_3_ = 0, *Z*_4_ = 1)
Monthly income (RMB)	1 = <500, 2 = 500~, 3 = 1,000~
Medical expenses/month	1 = <100, 2 = 100~, 3 = 500~
Medical insurance form	1 = Urban employee medical insurance, 2 = Urban and rural residents’ medical insurance
Disability level	1 = Mild disability, 2 = Moderate Disability, 3 = Severe Disability
Length of disability (years)	1 = 1~, 2 = 5~, 3 = 10~, 4 = 15~
Number of activities/rehabilitation exercises	1 = Never, 2 = Once a month, 3 = 2 ~ 4 times a month, 4 = 2 ~ 3 times a week, 5 = More than 4 times a week
Activity/rehabilitation exercise hours/time	1 = Never, 2 = Under 30 min, 3 = 30 min~, 4 = 1 h~, 5 = 2 h~
Degree of family support for the disabled older adults to participate in social activities	1 = Highly unsupportive, 2 = Unsupportive, 3 = Normal, 4 = Supportive, 5 = Highly supportive

**Table 4:** Multiple regression analysis results on the level of active aging of rural disabled older adults.

Variable		Partial regression coefficient	Standard error	Standardized regression coefficient	*t*	*P*
Constant		60.855	9.747	–	6.244	<0.001
Economic sources “Retirement pay” as the control group	Child support	−13.176	2.854	−0.453	−4.617	<0.001
	Government relief	−17.828	2.818	−0.497	−6.327	<0.001
	Pension	−11.847	2.918	−0.438	−4.059	<0.001
	Other (part-time work, farming, etc.)	−7.494	2.959	−0.179	−2.532	<0.001
Disability level		−5.480	0.809	−0.300	−6.771	<0.001
Activity/rehabilitation exercise hours/time		4.404	0.939	0.355	4.691	<0.001

## 4. Discussion

The active aging of rural older adults with disability is at a low level, and the level of economic security and social participation need to be improved. Economic source, disability level, and activity/rehabilitation exercise hours/time are the influencing factors of the level of active aging of rural older adults with disability (*p* < 0.05).

The total score of 67.38 (SD 13.11) for active aging among rural older adults with disability is lower than the findings of Huiying et al. (24) for rural older adults, which may be related to the fact that the rural older adults with disability surveyed have reduced activities of daily living (ADLs) and all suffer from different conditions and numbers of chronic diseases, mostly hypertension, neurological diseases and joint pain, which to a certain extent reduce the level of active aging among the older adults with disability. The health and community mobility of older adults is somewhat reduced, limiting the level of active aging (25). Xuelian et al. (26) demonstrated that declining ADL reduces active aging in older adults, that depression is a mediating influence, and that comorbid depression and declining ADL have a cumulative effect on active aging. Siltanen et al. (27) found lower levels of active aging in older adults with mobility impairments and that psychological flexibility could mitigate the negative effects of early walking difficulties on active aging. Furthermore, health literacy can help older adults with disability cope with illness and functional limitations and maintain a high level of active aging (28). Diverse applications of information and communication technology (ICT) can reduce isolation and enhance social participation of the older adults with disability. However, in the process of urbanization, the investment and resource distribution of senior care in rural areas are lower than those in cities, and the traditional perception of senior care makes the lack of organizational atmosphere for social participation of older adults in rural areas (29), the lack of active health behavior of disabled older adults and the prominent phenomenon of digital divide (30), which seriously restrict the process of active aging in rural areas. This suggests that we should build a rural and aging-appropriate information technology platform according to the current situation of electronic health literacy of the disabled older adults in rural areas, and integrate virtual reality and augmented reality technologies to conduct health lectures, train memory or perform rehabilitation exercises to increase the health knowledge reserve of the disabled older adults and enhance their dynamic interaction with the surrounding environment (31).

The highest score of 2.29 (SD 0.61) for the being self-reliant dimension of rural older adults with disability is consistent with the findings of Celmira et al. (32) and Hongjie et al. (33) for rural older adults, which may be related to the fact that 72.0% of the surveyed older adults are in a mild state of disability, and the lack of adequate human resources for the disabled due to the shortage of family caregivers in rural areas, coupled with the fact that the disabled older adults do not want to bring financial and caregiving burdens to their families, they still actively try to cope with daily activities independently and insist on doing the work they can. The lowest score of the active contribution to society (Mean 1.37, SD 0.55) dimension indicates that the social participation of the older adults with disability in rural areas is poor. The reason for this may be that the physical functional impairment of the older adults with disability is very likely to make them have low self-esteem and even close themselves off, avoiding or refusing social interactions, decreasing the mobility of their living space, and feeling that they have no experience and skills to pass on to others (34). Sariyamon et al. (35) found that the level of community friendly environment and healthy lifestyles were contributing factors to active aging among older adults. Two community empowerment factors, leisure and welfare facilities for older adults and cooperative alliances, were significantly associated with active aging (36). This suggests that village councils should actively create a caring community environment for the older adults, such as age-friendly renovation of neighborhoods, improving the hygiene of outdoor spaces (37), integrating multiple resources to provide regular home visits, or creating virtual health and age-friendly environments based on ICT to reduce social isolation and promote social participation (38).

The economic source was an influential factor in the level of active aging of rural disabled older adults, consistent with the findings of Huiying et al. (24). The highest level of active aging (*p* < 0.05) was found among the disabled older adults with retirement pay, who were mostly village workers returning to their hometowns, with higher education and monthly income overall, better health literacy and active health behaviors, and with sufficient financial security to reduce the psychological stress and financial burden of the disabled older adults, supporting their access to better material and health services and promoting a positive mindset toward social participation (39, 40). But only 7.6% of the rural disabled older adults had a retirement salary, and their main sources of financial support were still pension (37.2%), child support (28.9%) and government relief (15.8%), which indicates that the national pension welfare policy has gradually become the main financial security for the rural disabled older adults. Rural older adults with disability who relied on part-time work and farming to generate income accounted for 10.5% of the total, and their active aging scores were higher than those on government relief (*p* < 0.05). This may be related to the better overall physical and psychological health of the disabled older adults who engage in productive participation (41). However, the average score of building up financial security dimension for rural older adults with disability was only 1.37 (SD 0.57), with 63.5% of the older adults with disability had a monthly income of less than 500 (RMB), but 24.7% of the older adults with disability had monthly medical expenses of more than 500 (RMB), and their financial income microblogging and inability to make ends meet were obvious, and their overall financial security was not sufficient. This can be attributed to the weakened self-working capacity of the disabled older adults, reduced productive participation and economic income generation (14), weakened economic contribution to the family, and increased levels of depressive symptoms, which can easily lead to negative changes in aging attitudes (42). The national government should accelerate the implementation of long-term care insurance and medical assistance insurance for major diseases in rural areas, provide re-employment opportunities for the older adults with the ability to participate in production from the perspective of poverty alleviation, organize rural third-age universities to teach health knowledge (43), and gradually improve and implement the national welfare policy for the older adults.

The degree of disability was a negative predictor of the level of active aging among rural disabled older adults (*p* < 0.05), consistent with the results of the analysis by Hairu et al. (44). Decreased self-care can be a barrier to daily living and socialization, causing a passive reduction in the level of social participation and even negative emotions in older adults (45). A study by Sini et al. (46) confirmed that decreasing social distance reduces the chances of older adults to lead an active life. In this study, 69.4% of the older adults had been disabled for less than 5 years, 49.3% had become disabled within the last year, and 81.3% were disabled due to illness. The physical status of the older adults with short-term disability is still in the recovery stage, their physiological, role and self-concept adaptation levels are low, their overall health status is poor, and their healthcare needs are diverse (47). However, rural primary health care resources are limited, and home-bound disabled older adults only enjoy basic public health services and lack professional guidance and treatment at the health level, which to a certain extent affects their level of active aging (48). This indicates that the level of comprehensive primary health care in rural areas needs to be improved. Consider using the “Internet+” channel to help push active aging, promote the sinking of quality medical resources through medical associations/medical communities, and integrate multiple resources to provide personalized consultation and health promotion services (49). Reyhane et al. (50) provided a six-week health education component (once a week) on nutrition, physical activity, responsibility, stress management, communication and spirituality to older adults, demonstrating that training based on a health-enhancing approach can be effective in promoting active aging in older adults.

The longer the duration of each activity/rehabilitation exercise, the higher the level of active aging in rural older adults with disability, and 2–3 exercises per week for 1 h and more was a better activity/rehabilitation dose (*p* < 0.05). Sport/rehabilitation exercise is a health-level social participation that increases contact and communication with the outside world, and prolonged, regular exercise can help older adults with disability to improve their physical fitness, prevent or mitigate the negative effects of disease, and improve cognitive function (51, 52). Moreover, older adults with disability who actively participate in village activities or rehabilitation exercises tend to have better health awareness and active social participation, and higher levels of subjective well-being (53). However, there are still 37.2% of the disabled older adults never participate in activities and rehabilitation exercises, which may be related to the older adults’ fear of falling, and 47.7% of the surveyed disabled older adults’ family members do not support their participation in social activities, coupled with the low level of professional treatment in rural areas and the lack of rehabilitation specialists in rural health centers, resulting in the inability of the disabled older adults to obtain professional rehabilitation guidance and medical assistance. The negative impact on the participation of the older adults in activities (54). It suggests that grassroots governments should strengthen the construction of village activity facilities, actively mobilize the participation of multiple subjects such as village committees, kindergartens and volunteer associations, and organize multimodal social activities through channels such as jitterbug, radio and village stages (55). Silvia et al. (56) used new technology to create “scavenger hunts” to promote physical activity, where participants use a receiver GPS to hide or find real or virtual objects, adding gamification to self-tracking to promote participation and active aging in older adults. Furthermore, the role of “health mentors” can be established in villages to enhance the active aging of rural older adults with disability through the establishment of senior learning communities and intergenerational-based learning.

## 5. Conclusion

Rural older adults with disability have a low level of active aging and insufficient financial security and social participation. Rural older adults with moderate to severe disability are a key concern for the government in actively addressing population aging. The national government should actively improve older adult welfare policies to enhance economic security, promote the construction of medical associations/medical communities to improve the quality of rural primary health care, create a virtual senior community-friendly environment based on ICT, and innovate multi-modal physical exercise activities to promote social participation of the disabled older adults. However, this study only selected disabled older adults from rural in Henan Province, and the sample size was limited and not representative of the overall level of the disabled older adults in rural areas. Additionally, the questionnaire was completed with the help of the researcher to understand the subjective perceptions of the respondents, and some items such as “chronic diseases suffered” may be biased due to the respondents’ lack of knowledge about the diseases.

## Data availability statement

The original contributions presented in the study are included in the article/Supplementary material, further inquiries can be directed to the corresponding author.

## Ethics statement

The study was approved by the Life Science Ethics Review Committee of Zhengzhou University (ZZUIRB2021–155) and conducted in accordance with the Code of Ethics of the World Medical Association (Declaration of Helsinki). This survey was voluntary, anonymous and completely confidential. The older adults were informed that submitting the completed questionnaire was considered implied consent.

## Author contributions

YTT: investigation, data curation, formal analysis, writing - original draft, and writing – review and editing. YZ: conceptualization, resources, methodology, Writing – review and editing, and funding acquisition. YWY: investigation, formal analysis, visualization. HZZ: investigation and data curation. XZL: data curation and formal analysis. All authors contributed to the article and approved the submitted version.

## Funding

Funding support was provided by the National Natural Science Foundation of China (71874162).

## Conflict of interest

The authors declare that the research was conducted in the absence of any commercial or financial relationships that could be construed as a potential conflict of interest.

## Publisher’s note

All claims expressed in this article are solely those of the authors and do not necessarily represent those of their affiliated organizations, or those of the publisher, the editors and the reviewers. Any product that may be evaluated in this article, or claim that may be made by its manufacturer, is not guaranteed or endorsed by the publisher.
